# The Long-Term Effect of Kidney Transplantation on the Serum Fatty Acid Profile

**DOI:** 10.3390/nu16193319

**Published:** 2024-09-30

**Authors:** Maciej Śledziński, Justyna Gołębiewska, Adriana Mika

**Affiliations:** 1 Department of General, Endocrine and Transplant Surgery, Faculty of Medicine, Medical University of Gdansk, 80-214 Gdansk, Poland; msledz@gumed.edu.pl; 2 Department of Nephrology, Transplantology and Internal Medicine, Medical University of Gdansk, 80-211 Gdansk, Poland; justyna.golebiewska@gumed.edu.pl; 3 Department of Pharmaceutical Biochemistry, Faculty of Pharmacy, Medical University of Gdansk, 80-211 Gdansk, Poland; 4 Department of Environmental Analytics, Faculty of Chemistry, University of Gdansk, 80-308 Gdansk, Poland

**Keywords:** fatty acids, kidney transplant, end-stage kidney disease, nutrition

## Abstract

**Background:** Epidemiologic evidence has demonstrated the prevalence of metabolic disorders and increased cardiovascular risk related to lipid metabolism disorders in kidney transplant recipients. Therefore, it is of great importance to understand lipid alterations and to look for ways to reduce cardiovascular risk in this patient group. **Methods:** Our study included 25 patients with chronic kidney disease undergoing kidney transplantation (KTx). Three blood samples were taken from each patient: before KTx, 3 months after KTx and 6–12 months after KTx. A series of biochemical blood tests and a detailed analysis of the serum fatty acid profile were performed. **Results:** In our previous study, the effects of kidney transplantation on serum fatty acid (FA) profile 3 months after the procedure were investigated. The current study shows the longer-term (6–12 months) effects of the procedure on the serum FA profile. We found that although *n*-3 polyunsaturated FA levels started to decrease 3 months after surgery, they normalized over a longer period of time (6–12 months). Furthermore, we observed a strong decrease in ultra-long-chain FAs and an increase in odd-chain FAs over a longer time after kidney transplantation. All of the above FAs may have an important impact on human health, including inflammation, cardiovascular risk or cancer risk. **Conclusions:** The changes in serum FA profiles after kidney transplantation are a dynamic process and that more detailed studies could provide an accurate indication for supplementation with some FAs or diet modification.

## 1. Introduction

Patients with chronic kidney disease (CKD) face an increased mortality rate and an increased risk of cardiovascular disease (CVD) due to dyslipidemia [1]. Kidney transplantation (KTx) offers a better quality of life but still carries metabolic risks, including cardiovascular problems [2].

Fatty acid (FA) imbalance plays a crucial role in these complications, as CKD can disrupt FA profiles. *N*-3 polyunsaturated fatty acids (*n*-3 PUFAs) are known for their cardiovascular benefits, such as reducing oxidative stress and inflammation [3,4]. Monounsaturated FAs (MUFAs), in turn, are associated with various metabolic diseases including CKD [5]. In CKD patients, serum MUFAs are increased and *n*-3 PUFAs are decreased, which is associated with an increased risk of CVD. Most studies focus on the effects of supplementation with *n*-3 PUFAs on renal function and lipid profiles [6,7]. Research on the FA profile in kidney transplant recipients is limited.

Our team’s studies, comparing transplant recipients with healthy individuals, found that the levels of potentially beneficial *n*-3 PUFAs in patients at different time points after KTx (1–12 months) are reduced [8]. These results suggest that even with normal kidney function after transplantation, the FA profile remains abnormal. It is unlikely that this is solely due to a change in diet but may also be due to the effects of immunosuppressants or other unknown causes. These significant disturbances in the FA profile may increase the risk of CVD in this susceptible patient group [9,10]. 

To determine more precisely the changes in FA profile after transplantation, in another study we examined the FA profile of the same patients shortly before and 3 months after KTx [11]. This study revealed changes in the FA profile of CKD patients 3 months after KTx. The most notable changes included a significant reduction in *n*-3 PUFAs. We also observed a decrease in ultra-long-chain FAs with 26 or more carbon atoms and an increase in the desaturation index (C18:1/C18:0) after transplantation [11].

A significant decrease in the concentration of *n*-3 PUFAs in renal transplant recipients 3 months after transplantation may contribute to an increased risk of CVD. Patients recover in the first 3 to 4 months after a KTx, and the risk of kidney rejection is highest during this time. Stronger immunosuppression is used during this period. However, after this critical period, the patient’s condition stabilizes [12] and the intensity of immunosuppressive therapy decreases, potentially influencing further changes in lipid metabolism, including the FA profile. It seems necessary to evaluate the changes in the FA profile in the same group of patients over a longer period after transplantation. Long-term assessment of FA profile changes is crucial as metabolic changes develop gradually and may remain undetected in short-term studies. Further analysis may reveal unknown FA changes that impact patient health, including inflammation, cardiovascular risk and metabolic balance. Fatty acids are crucial for the regulation of inflammation and lipid homeostasis, and an imbalance could increase the risk of cardiovascular disease, metabolic disorders or even cancer. Therefore, studying FA profiles over time can provide insights for interventions such as nutritional supplementation or dietary adjustments to improve long-term health outcomes. The aim of this study was to investigate the changes in the FA profile over a longer period after KTx (6–12 months).

## 2. Materials and Methods

### 2.1. Patients

The study included 25 CKD patients who underwent KTx at the University Clinical Center in Gdansk. Of these, 23 patients received kidneys from deceased donors, and 2 from living donors. Written informed consent for the study was obtained upon enrollment. Permission for this study was obtained from the independent Bioethics Committee of the Medical University of Gdansk (protocol No. NKBBN/512-358/2021). 

Patients who had not consented to participate in the study and patients taking FA supplements were excluded. For each of the 25 patients, 3 blood samples were taken at 3 different time periods—before KTx, 3 months after KTx and 6–12 months after KTx. In the previous study [11], 35 patients were included. In this study with longer follow-up, this number decreased because some patients did not return for follow-up (migration) or lost their transplanted kidney and had to undergo dialysis again. Kidney transplant recipients were admitted to the hospital after being selected from the waiting list. On the day of the transplant, the transplant team removed the kidney from the donor. A fasting blood sample was then taken from the CKD patients. After centrifugation at 3000× *g*, the serum sample was coded and stored at −80 °C for further FA analysis. The timing of the blood sampling at the second follow-up point resulted from the fact that the patients appeared for their third examination at different times. Some of them came from different cities and could not appear at a fixed time for the third examination.

After successful transplantation, patients received nutritional instructions according to Polish guidelines [13]. Then, 3 and 6–12 months after transplantation, blood samples were taken again during routine check-ups at the Nephrology Clinic and the serum was stored in the same way as on the day of transplantation.

### 2.2. Fatty Acid Analysis

The FA profile was assessed in serum samples collected at three different time points by the gas chromatography–mass spectrometry (GC–MS) method described in our previous study [11]. In brief, total lipids were extracted from the tissue samples using a chloroform–methanol mixture (2:1, *v*/*v*). After drying under nitrogen stream, the extracted lipids were hydrolyzed with 0.5 M KOH at 90 °C for 3 h. After incubation, the mixtures were acidified with 6 M HCl, and 1 mL of water was added. The non-esterified fatty acids were extracted three times with 1 mL *n*-hexane and the organic phase was evaporated under nitrogen stream. The extracted FAs were then derivatized with 10% boron trifluoride in a methanol solution at 55 °C for 1.5 h to give FA methyl esters (FAMEs). Then, 1 mL of water was added and the FAMEs were extracted with *n*-hexane (3 × 1 mL), dried under nitrogen and stored at –20 °C until analysis.

FAMEs were analyzed using a GC-EI-MS QP-2020 NX (Shimadzu, Kyoto, Japan) with chromatographic separation on a Zebron ZB-5MSi capillary column, 30 m × 0.25 mm i.d. × 0.25 μm film thickness, (Phenomenex, Torrance, CA, USA). Samples were injected in split mode using dichloromethane as solvent. The column temperature was set in a range from 60 °C to 300 °C at a rate of 4 °C/min, with helium as carrier gas and a column head pressure of 60 kPa. The temperature of the injection, ion source and transfer line were 300 °C. The ionization of FAMEs was performed by electron ionization at 70 eV. 19-methylarachidic acid was used as internal standard. The full scan mode with a mass scan range of *m*/*z* 45 to 700 was used. Accurate identification of the FA profile was possible based on FAME mixture standards (Larodan, Michigan, USA and Merck, Darmstadt, Germany).

### 2.3. Statistical Analysis

Data analysis was performed using Sigma Plot 14.5 software (Systat Software Inc., San Jose, CA, USA). The Shapiro–Wilk test was used to assess the normality of the data distribution. Groups were compared using repeated measures one-way ANOVA with Bonferroni correction across three time points. The results are presented as mean and standard deviation (SD). Statistical significance was set at *p* < 0.05. The figures were generated in Excel Microsoft 365. To assess the quality of the results, we performed post hoc power calculations for each parameter evaluated. All statistically significant differences reached a power level greater than 0.8.

## 3. Results

The characteristics of the study group are shown in Table 1. For 19 patients it was the first transplantation, for 4 it was the second and for 2 the third. The panel of reactive antibodies (PRA) was 0% in 20 patients before transplantation. Four patients had PRA ≤ 20% and one had 47%. The mean number of mismatches for the six antigens examined in this group was 3.04 ± 1.31. Nineteen patients had no donor-specific antibodies (DSAs), while the remaining six patients had low DSA levels (<3000). Delayed graft function (DGF) occurred in seven patients after transplantation. None of the patients in this current study group experienced rejection during the 12-month period. In addition, none of the patients required a change in immunosuppressive medication or an increase in dosage.

A total of nine patients were taking statins up to the time of KTx. None of the patients took these drugs in the first 3 months after KTx. After the third month, two patients resumed taking atorvastatin. 

Comparison of laboratory results before transplantation with those taken 3 and 6 to 12 months after transplantation shows a significant improvement in several parameters related to kidney function. Creatinine levels decreased and estimated glomerular filtration rate (eGFR) increased. The increase in eGFR was also statistically significant between 3 and 6–12 months after KTx. Hemoglobin and sodium concentration increased and potassium concentrations decreased. The C-reactive protein (CRP) concentration, which serves as an indicator of the inflammatory status, decreased significantly. Over a longer period of time (6–12 months), the hemoglobin and creatinine concentrations reached the reference values (Table 1). Conversely, aspartate aminotransferase (AST) and alanine aminotransferase (ALT) concentrations tended to rise over a longer period of time after transplantation, which could be related to the toxic effects of the immunosuppressive drugs taken over a longer time.

**Figure 1:**
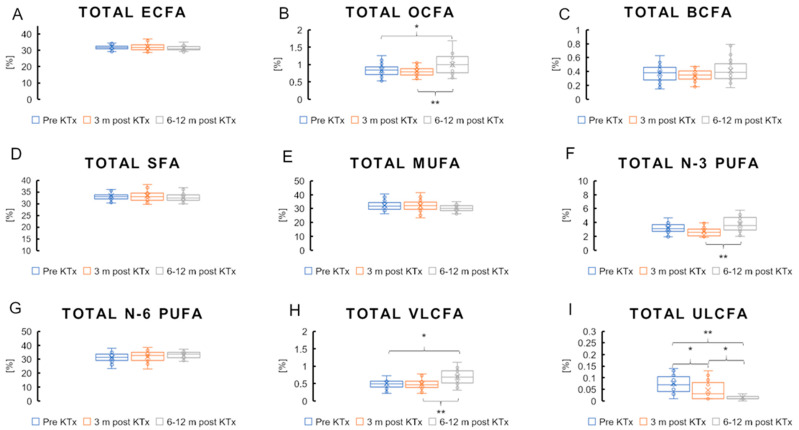
Fatty acid content (%) in patient serum. ECFAs—even-chain fatty acids. OCFAs—odd-chain fatty acids. BCFAs—branched-chain fatty acids. SFAs—saturated fatty acids. MUFAs—monounsaturated fatty acids. *n*-6 PUFAs—*n*-6 polyunsaturated fatty acids. *n*-3 PUFAs—*n*-3 polyunsaturated fatty acids. VLCFAs—very-long-chain fatty acids (20:0–32:0). ULCFAs—ultra-long-chain fatty acids (26:0–32:0). Values are mean ± SD. * Statistical significance (*p* < 0.05) and ** *p* < 0.001.

The total value of even-chain saturated FAs (ECFAs), odd-chain saturated fatty acids (OCFAs), branched-chain saturated fatty acids (BCFAs) and the total of saturated fatty acids (SFAs) did not change 3 months after transplantation in a previous study [11]. Even after 6–12 months, no changes were observed in these groups, with the exception of OCFAs, which increased significantly (Figure 1A–D). Table 2 shows the levels of some individual FAs among over 50 that were analyzed, along with which levels changed significantly during therapy. Among the ECFAs, the levels of FAs containing 14, 20, 22 and 24 carbon atoms were significantly higher 6–12 months after transplantation than before the procedure. The opposite was observed for palmitic acid (C16:0), the amount of which decreased (Table 2).

The total amount of very-long-chain FAs (VLCFAs) containing more than 20 carbon atoms did not change significantly 3 months after KTx but increased significantly after 6–12 months (Figure 1H; 0.50% vs. 0.48% vs. 0.67%; *p* < 0.05). The total amount of ultra-long-chain FAs (ULCFAs) containing 26 or more carbon atoms was significantly reduced 3 months after KTx, and this reduction was further deepened 6–12 months after surgery (Figure 1I; 0.089% vs. 0.052% vs. 0.024%; *p* < 0.05). The total amount of total MUFAs did not change after transplantation over a shorter and longer time interval (Figure 1E). Among the MUFAs, a significant increase was observed 6–12 months after transplantation for some FAs (C20:1, C22:1 and C24:1), while 18:1 showed a decreasing trend during this period (Table 2). Total *n*-3 PUFAs increased significantly 6–12 months after KTx after an initial downward trend 3 months after KTx (Figure 1F; 3.22% vs. 2.76% vs. 3.7; *p* < 0.05). The change in the amount of docosahexaenoic acid (DHA) initially showed a significant decrease at 3 months and then a significant increase at 6–12 months (Table 2). Some *n*-6 PUFAs behaved similarly: arachidonic acid (ARA) and dihomo-γ-linolenic acid (DGLA) decreased significantly 3 months after KTx and then increased significantly 6–12 months after KTx. Linoleic acid (LA), however, increased significantly in the shorter period after treatment (3 months), but after 12 months there is only a trend towards an increase in LA compared to the preoperative samples (Table 2). Total *n*-6 PUFAs did not change significantly either 3 or 6–12 months after KTx (Figure 1G).

## 4. Discussion

Comparing the results of this study, which included a 6–12-month follow-up, with our earlier study, which only referred to a 3-month observation period, it can be seen that a significant improvement in renal function is confirmed at both time points after transplantation. Long-term follow-up led to a higher increase in the GFR index and a further upward trend in creatinine and hemoglobin concentrations towards normalization of renal function parameters. In both shorter and longer follow-up periods after KTx, we have seen a reduction or trend towards a reduction in CRP levels, suggesting a reduction in inflammation in transplant patients. In the longer-term follow-up, there is also an increasing trend in liver enzyme concentrations, which could be related to immunosuppressive therapy [14,15].

In a previous study, we have shown that the total amount of *n*-3 PUFAs decreased after transplantation. In this study, the decrease in total *n*-3 PUFAs 3 months after transplantation was not significant, which may be related to the smaller number of patients included in the present study, but after an initial downward trend, the total amount of *n*-3 PUFAs increased significantly after 6–12 months. Some key *n*-3 PUFAs, such as DHA, were significantly lower 3 months after surgery and normalized after 6–12 months. These changes may be due to a change in diet. From 2019, after KTx, patients have received detailed nutritional recommendations based on Polish Guidelines [13] when they are discharged home. These recommendations are mainly based on Australian recommendations [16,17] and emphasize that immunosuppressive treatment increases existing fat and carbohydrate disorders, which increases the risk of obesity and infections. As maintaining body weight is most important, a diet with limited carbohydrates, reduced calories and reduced simple sugars is, therefore, recommended. If lipid metabolism disorders are detected, treatment of hyperlipidemia through lifestyle changes, diet and pharmacotherapy is indicated. A diet rich in whole grain products with a low glycemic index, products with a high fiber content, products rich in vitamin E and mono- and polyunsaturated fatty acids (vegetable oils, vegetables and proteins of vegetable rather than animal origin) is recommended. Patients are informed that they should supplement their diet with fish products [13]. Due to economic problems and the nature of the Polish diet, these products do not form a large part of the diet. Patients more often choose to take fish oil supplements. The most frequently increased dietary product among our patients is rapeseed oil, which is a source of LA [18]. The LA can be converted into longer and more desaturated *n*-6 PUFA in the human body. The study showed that LA levels increased significantly after 3 months and were maintained after 6–12 months. Since *n*-3 PUFAs are considered cardioprotective, and reduce oxidative stress and inflammation [19], their increase 6–12 months after KTx likely reduces CVD risk for longer after the procedure. In this work, we have also found that some *n*-6 PUFAs, such as ARA and DGLA, decreased after 3 months but increased significantly after 6–12 months. This increase in *n*-6 PUFAs, particularly ARA, may have the opposite effect to the increase in *n*-3 PUFAs, as ARA is a precursor of proinflammatory prostaglandins [19]. Our previous work focused primarily on changes observed 3 months after transplantation, whereas the current study examines changes at both 3 months and 6–12 months. This may explain some of the differences, particularly in cases where the changes are gradual and may only become imperceptible over a longer period of time. Changes in individual FA may be due to changes in patients’ lipid metabolism after KTx. Immunosuppressive therapy may have different effects on FA metabolism [20,21,22], which could explain the observed differences in the amounts of specific FA between different study periods after KTx. After 6–12 months, an increase in the total amount of OCFAs was observed, which could have a positive effect. OCFAs prevent and reduce the risk of CVD and inflammation [23]. The main source of OCFAs is ruminant meat and dairy products [24]. Pre-transplant patients have a limited protein intake due to kidney failure and at the same time a high protein requirement immediately after transplantation due to the metabolic stress associated with surgery and the use of high doses of steroid medication [13]. An increased protein intake is recommended during this period. Nevertheless, no increase in OCFAs is observed in the 3-month period after transplantation, which could indicate an increased consumption of protein sources other than dairy products and ruminant meat. In some patients, hypercalcemia is observed after KTx as a result of persistent secondary hyperparathyroidism [25], which may be the reason for restricting dairy products in the early post-transplant period. Over the next 6–12-month period, the amount of OCFAs increases significantly, which could indicate dietary expansion and minor restrictions related to calcium metabolism disorders. In many studies, pentadecanoic acid (C15:0) and heptadecanoic acid (C17:0) have been associated with positive health effects. C15:0 can exert an anti-cancer effect in breast cancer cells [26], lung, pancreas and liver in vivo [27] and promote an anti-inflammatory state in obese mice [28]. A high-fiber diet leads to increased C17:0 levels [29], which are associated with improved insulin sensitivity [30]. A diet high in dairy products increases C15:0, which also improves insulin sensitivity but to a lesser extent than C17:0 [31]. Our previous study [23] showed an inverse correlation between serum total OCFAs and CRP levels in obese subjects, suggesting the potential anti-inflammatory effect of OCFAs. The number of VLCFAs did not change significantly after 3 months, but increased significantly after 6–12 months, which could be related to poorer liver function as a result of immunosuppression. In both studies, a significant reduction in the number of ULCFAs with 26 or more carbon atoms was observed 3 months after transplantation. Interestingly, this decrease deepened even more in the longer observation period, in contrast to the amount of total VLCFAs. The amounts of ULCFAs are much lower than the total amounts of VLCFAs, which may lead to opposite results. High levels of 26:0 and other ULCFAs are associated with various CVD symptoms [32,33,34]. Thus, a reduction of ULCFAs could be a beneficial effect of KTx that prevents CVD [32].

The study was limited by the relatively small study group and the diversity of patients. The patients studied had different causes of kidney failure and were taking different medications, most commonly for high blood pressure, due to disorders in the course of the disease. Each patient had their own dietary habits, which were modified to a certain extent by recommendations on discharge from hospital after transplantation. It was not possible to objectively assess the composition of the diet of the patients studied using dietary questionnaires. However, the strength of this study is the ability to compare the FA profiles at three time points for each patient included in the study. The second follow-up was conducted at a different time interval (6–12 months) due to problems with patients’ concurrent attendance at the clinic after KTx.

## 5. Conclusions

The current study extends the observations of changes in serum FA profiles after KTx to a period of 6–12 months and reveals additional changes in lipid metabolism that were not visible in the first 3-month follow-up. Increased *n*-3 PUFAs levels and decreased ULCFAs levels represent an improvement in FA profiles that may reduce CVD risk over a longer post-transplant period. In summary, these findings highlight the dynamic nature of lipid metabolism after KTx, which could have significant implications for patient management and potential dietary or therapeutic interventions to optimize long-term outcomes.

## Figures and Tables

**Table 1:** Characteristics of the study group.

Parameters	Pre KTx	3 m Post KTx	6–12 m Post KTx	*p*			
				Pre vs. 3 m	Pre vs. 6–12 m	3 m vs. 6–12 m	Reference Value
	*n* = 25						
Age	50.3 ± 14.0						
Sex	M:13 F:12						
BMI (kg/m^2^)	25.7 ± 3.29	25.8 ± 3.23	26.2 ± 3.36	NS	NS	NS	18.5–24.9
Hemoglobin (g/dL)	11.2 ± 1.43	12.6 ± 2.26	13.4 ± 1.91	<0.05	<0.001	NS	13.0–17.0
eGFR-CKD (mL/min/1.73 m^2^)	7.5 ± 3.35	49.8 ± 15.1	58.5 ± 15.6	<0.001	<0.001	<0.05	>60
CRP (mg/L)	7.9 ± 9.1	2.9 ± 6.6	2.7 ± 3.1	<0.05	0.057	NS	0.0–5.0
Creatinine (mg/dL)	8.0 ± 2.8	1.5 ± 0.4	1.3 ± 0.3	<0.001	<0.001	NS	0.7–1.3
Sodium (mmol/L)	139 ± 2.24	141 ± 2.52	141 ± 2.70	NS	NS	NS	136–145
Potassium (mmol/L)	4.7 ± 0.1	4.4 ± 0.5	4.3 ± 0.5	<0.05	<0.05	NS	3.5–5.1
ALT (U/L)	20.5 ± 12.0	28.6 ± 18.5	51.8 ± 49.79	NS	<0.05	NS	<55
AST (U/L)	21.2 ± 13.4	22.8 ± 15.8	41.6 ± 38.32	NS	NS	NS	5–34
TG (mg/dL)	212 ± 151	196 ± 92	160 ± 130	NS	NS	NS	<150
CHOL (mg/dL)	194 ± 52	232 ± 39	176 ± 28	NS	NS	NS	115–190
	Immunosuppressive drugs						
MP + Tc + MM				n = 21			
MP + Cc + MM				n = 3			
MP + Tc				n = 1			
renal replacement therapy before KTx							
Peritoneal Dialysis *n* = 6 Hemodialysis *n* = 17							

Pre KTx—just before KTx. m post KTx—months after KTx. ALT—alanine aminotransferase. AST—aspartate aminotransferase. BMI—body mass index. Cc—ciclosporin. CHOL—total cholesterol. CRP—C-reactive protein. eGFR-CKD—estimated glomerular filtration rate using CKD-EPI creatinine equation. NS—nonsignificant. MP—methylprednisolone. MM—mycophenolate mofetil. Tc—tacrolimus. TG—triacylglycerols. Values are presented as a mean ± SD.

**Table 2:** The fatty acid changes during therapy and the content (%) in patient serum.

FA (%)	Pre KTx	3 m Post KTx	6–12 m Post KTx		*p*	
				Pre vs. 3 m	Pre vs. 6–12 m	3 m vs. 6–12 m
C14:0	1.20 ± 0.34	1.46 ± 0.60	1.51 ± 0.41	NS	0.031	NS
C16:0	22.7 ± 1.49	23.2 ± 2.11	20.8 ± 2.01	NS	<0.001	<0.001
C17:0	0.31 ± 0.053	0.30 ± 0.050	0.37 ± 0.093	NS	NS	0.008
C19:0	0.020 ± 0.009	0.020 ± 0.007	0.033 ± 0.010	NS	<0.001	<0.001
C20:0	0.12 ± 0.042	0.13 ± 0.038	0.20 ± 0.086	NS	<0.001	<0.001
C21:0	0.020 ± 0.014	0.019 ± 0.008	0.032 ± 0.016	NS	0.024	0.010
C22:0	0.21 ± 0.085	0.21 ± 0.068	0.32 ± 0.095	NS	0.024	0.010
C23:0	0.066 ± 0.028	0.077 ± 0.030	0.122 ± 0.049	NS	<0.001	<0.001
C24:0	0.22 ± 0.08	0.23 ± 0.08	0.33 ± 0.09	NS	<0.001	<0.001
C25:0	0.052 ± 0.031	0.028 ± 0.020	0.016 ± 0.011	<0.001	<0.001	0.074
C26:0	0.031 ± 0.021	0.020 ± 0.010	0.014 ± 0.008	0.021	<0.001	NS
C30:0	0.022 ± 0.010	0.009 ± 0.007	traces	NS	<0.001	<0.001
C32:0	0.013 ± 0.005	0.004 ± 0.004	traces	NS	<0.001	<0.001
iso C16:0	0.049 ± 0.017	0.054 ± 0.021	0.077 ± 0.033	NS	<0.001	0.002
iso C17:0	0.11 ± 0.030	0.10 ± 0.026	0.11 ± 0.050	NS	NS	0.051
C14:1	0.05 ± 0.03	0.08 ± 0.05	0.08 ± 0.06	NS	0.060	NS
C18:1	28.5 ± 2.73	28.0 ± 3.62	26.6 ± 3.55	NS	0.052	NS
C20:1	0.14 ± 0.052	0.17 ± 0.078	0.25 ± 0.104	NS	<0.001	0.004
C22:1	0.022 ± 0.013	0.022 ± 0.010	0.030 ±0.016	NS	0.049	0.065
C24:1	0.29 ± 0.11	0.32 ± 0.15	0.46 ± 0.19	NS	<0.001	<0.001
ETA (20:4 *n*-3)	0.076 ± 0.024	0.065 ± 0.034	0.107 ± 0.042	NS	0.020	<0.001
EPA (20:5 *n*-3)	0.82 ± 0.22	0.79 ± 0.42	1.05 ± 0.38	NS	0.052	0.022
DPAn3 (22:5 *n*-3)	0.45 ± 0.094	0.39 ± 0.084	0.48 ± 0.11	0.057	NS	0.005
DHA (22:6 *n*-3)	1.55 ± 0.44	1.21 ± 0.43	1.65 ± 0.63	0.010	NS	<0.001
LA (18:2 *n*-6)	23.0 ± 3.21	25.3 ± 3.96	24.5 ± 2.75	0.003	0.054	NS
EDA (20:2 *n*-6)	0.12 ± 0.040	0.13 ± 0.032	0.20 ± 0.056	<0.001	<0.001	NS
DGLA (20:3 *n*-6)	1.20 ± 0.39	0.95 ± 0.36	1.32 ± 0.34	0.009	NS	<0.001
ARA (20:4 *n*-6)	6.45 ± 1.03	5.06 ± 0.90	6.25 ± 1.29	<0.001	NS	<0.001
AdA (22:4 *n*-6)	0.16 ± 0.039	0.13 ± 0.045	0.18 ± 0.061	NS	NS	0.001

Pre KTx—just before kidney transplant surgery. m post KTx—months after kidney transplantation. LA—linoleic acid (18:2 *n*-6), EDA—eicosadienoic acid (20:2 *n*-6), DGLA—dihomo-γ-linolenic acid (20:3 *n*-6), ARA—arachidonic acid (20:4 *n*−6), AdA—adrenic acid (22:4 *n*-6), ETA—eicosatetraenoic acid (20:4 *n*−3), EPA—eicosapentaenoic acid (20:5 *n*-3), DPAn3—docosapentaenoic acid (22:5 *n*-3) and DHA—docosahexaenoic acid (22:6 *n*-3). NS—nonsignificant.

## Data Availability

The original contributions presented in the study are included in the article, further inquiries can be directed to the corresponding author.
